# Varicella Zoster Meningitis, Optic Neuritis Preceding the Development of Posterior Outer Retinal Necrosis, and Central Retinal Artery Occlusion in a HIV Patient

**DOI:** 10.1155/2019/4213162

**Published:** 2019-07-28

**Authors:** Manasa Gunturu, Shiva Kumar Gosi, Swetha Kanduri, Vishnu Garla

**Affiliations:** ^1^Department of Neuro-Ophthalmology, Bascom Palmer Eye Hospital, Miami, FL, USA; ^2^Department of Hospital Medicine, Banner Thunderbird Medical Center, Glendale, AZ, USA; ^3^Department of Internal Medicine, University of Mississippi Medical Center, Jackson, MS, USA

## Abstract

Varicella-zoster virus (VZV) has been known to cause various eye disorders in both immunocompetent and immunocompromised patients. We present a case of a forty-nine-year-old female patient with acquired immunodeficiency syndrome (AIDS) who presented with headache, fever, and blurred vision. Cerebrospinal fluid (CSF) analysis was consistent with VZV meningitis. Magnetic resonance imaging (MRI) of the brain showed enhancement of the right optic nerve indicative of optic neuritis. She responded well to acyclovir and steroids and discharged on the same. Four weeks after discharge, she presented with sudden onset blindness in the left eye. A cerebral angiogram revealed left retinal artery occlusion and was treated with tissue plasminogen activator (tPA). Funduscopic examination showed patchy areas of necrosis in the periphery which were rapidly progressive, diagnostic of posterior outer retinal necrosis (PORN). She was started on ganciclovir and cidofovir and experienced significant improvement in her visual acuity.

## 1. Introduction

VZV is one of the most common pathogens affecting the eyes in patients with human immunodeficiency virus (HIV). Initially, it was thought to be a harbinger of AIDS, but now it is being increasingly recognized in every stage of AIDS. VZV can cause a myriad of neurological and ophthalmological complications including meningitis, retinitis, optic neuritis, and retinal artery occlusion [1]. PORN was first described by Forster et al. in 1990. PORN is seen in severely immunocompromised patients. It is characterized by rapid infection of the outer layers of the retina with absence of intraocular inflammation and poor response to acyclovir therapy [2]. Central artery retinal artery occlusion (CRAO) due to VZV has been reported in the literature in nine instances with the first one being reported in 1920 [3, 4]. It is presumed that granulomatous angiitis of the involved artery may be the mechanism involved. It is probably one of the underdiagnosed causes of blindness associated with VZV [4].

We present a case of VZV meningitis, optic neuritis preceding the development of PORN, and CRAO in a HIV patient. To our knowledge, this is the first case of all these rare complications affecting the same patient.

## 2. Case Presentation

A 49-year-old African American female patient with a history of untreated HIV infection presented to the emergency room with a two-week history of left ear pain, headache, fever, and blurred vision. Physical exam was significant for crusted vesicles in the left ear and a vesicular skin rash on the left side of the face consistent with herpes zoster. Ophthalmic examination showed normal visual acuity (VA) in the left eye. VA in the right eye was decreased to 20/100 and an afferent pupillary defect in the right eye was also observed. The fundoscopic examination was unremarkable bilaterally. CSF analysis revealed lymphocytosis consistent with viral meningitis. VZV polymerase chain reaction (PCR) was positive confirming VZV meningitis. She was started on intravenous acyclovir (Table 1). MRI of the brain revealed enhancement of the right optic nerve and chiasm consistent with retrobulbar optic neuritis. Laboratory assessment showed a low cluster of differentiation 4 (CD4) count and high HIV viral load consistent with uncontrolled AIDS (Table 1). The patient was started on tenofovir, emtricitabine, and boosted darunavir for AIDS management and on high-dose methylprednisolone for optic neuritis. She experienced clinical resolution of her fever and headache. She was discharged on valacyclovir and prednisone taper.

**Figure 1:**
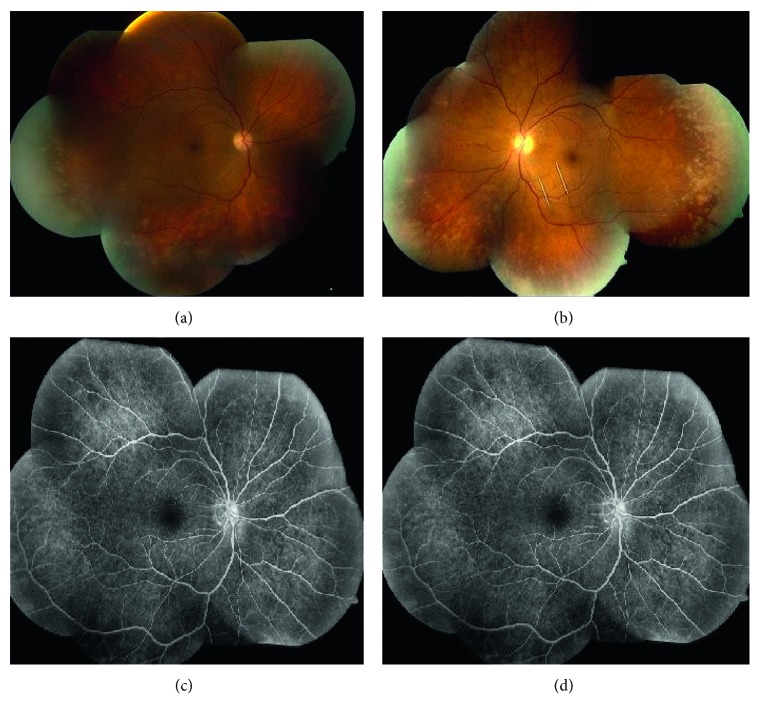
(a, b) Multiple discrete yellow-white chorioretinal lesions in the peripheral retina circumferentially which are more confluent in the far periphery. (c, d) Stippled areas of hyperfluorescence and hypoautofluorescence, both indicating abnormal retinal pigment epithelium.

Four weeks later, the patient presented to the emergency room with sudden painless loss of vision in her left eye. A cerebral angiogram revealed stenosis of the left ophthalmic artery. tPA was administered which resulted in an improvement in her visual acuity from 20/400 to 20/100 in her left eye. She was started on acyclovir and steroids. Fundoscopic examination revealed the progression of the necrosis ultimately involving the whole retina (see Figure 1). Fluorescein angiography was consistent with PORN. PCR performed on the vitreal fluid was positive for VZV. Intraocular ganciclovir and IV cidofovir were started to help prevent the progression of PORN. Laser walling was done subsequently to prevent retinal detachment.

The patient responded well to the antiretroviral treatment, her CD4 count six months later was 317 cells/cubic milliliter, and her viral load decreased to 50 copies/ml. Her visual acuity after one year showed significant improvement to 20/40 bilaterally.

## 3. Discussion

VZV is a DNA virus belonging to the herpes virus family. Primary infection with VZV results in varicella while secondary reactivation results in herpes zoster. Complications of VZV infection can occur in both the immune competent and immune compromised patients. However, it has been observed that involvement of multiple cranial or cervical dermatomes, extensive skin involvement, and severe pain occur more frequently in the immune compromised population [5, 6].

Herpes zoster ophthalmicus is due to reactivation of the virus in trigeminal ganglion and can result in a variety of ocular manifestations (Table 2) [7]. Optic neuritis is the inflammation of the optic nerve. Optic neuritis in HIV can be secondary to a number of causes such as syphilis, toxoplasmosis, histoplasmosis, central nervous lymphoma, cytomegalovirus, and HIV microvasculopathy [8]. It was first described by Hutchinson in 1866. Optic neuritis after VZV meningitis can be due to the direct invasion of the optic nerve by the virus from the sinus cavernosus. There are reports of virus particles isolated in the retina and optic nerve in a patient who died of herpetic encephalitis [9]. The other mechanism is an occlusive thrombotic granulomatous reaction set off in the vascular wall by the viruses or immune complexes. A case report of zoster-induced vasculitis of posterior ciliary artery producing optic neuritis has been documented in the literature [10]. Optic neuritis is characterized by decreased visual acuity, an afferent pupillary defect with a normal funduscopic examination. It is often preceded by onset of herpes zoster skin infection. MRI is the imaging modality of choice and may show enhancement of the affected optic nerve. Optimal treatment includes two weeks of high-dose intravenous acyclovir followed by two weeks of oral acyclovir [11].

VZV retinitis comprises two different entities, acute retinal necrosis (ARN) and PORN. ARN can occur in both immunocompetent and immunocompromised persons and is characterized by vitiris, choroidal vasculitis, and full-thickness retinal necrosis starting peripherally. PORN is seen only in immunocompromised persons and is characterized by multifocal retinal necrosis without granular borders in the deeper retinal layers which start peripherally and rapidly involved the whole retina. PORN is usually seen in severely immunocompromised patients with AIDS and can be confirmed by performing a VZV PCR on the intravitreal fluid [12]. Ormerod et al. studied eleven patients (twenty eyes) with PORN and noted that bilateral involvement, rapid progression, and relative resistance to acyclovir were common. 70% had retinal detachment and 70% had no light perception by the end of the study [13]. Sittivarakul and Aui-aree analyzed eleven eyes with PORN and noted 57% with bilateral involvement and 54% with retinal detachment. Prompt diagnosis and management of PORN is essential to limit damage. Visual prognosis is extremely poor in these cases with studies suggesting that progression to no light perception occurs within 1–6 months [14].

Retinal artery occlusion secondary to herpes zoster has been described in nine instances in the literature. The age range of these patients varied between 58 and 77 years, and the incubation period varied between five weeks to two years. The mechanism is similar to herpes zoster associated cerebrovascular accidents, through the formation of granulomatous angiitis. Studies have shown zoster viral particles in the media of the arteries in postmortem biopsies. It has been proposed that the virus travels from the trigeminal ganglion travels to the internal carotid artery and its branches via the sympathetic nerves and can cause CRAO [14].

Franco-Paredes et al. have described eight cases of VZV retrobulbar optic neuritis preceding retinitis in AIDS patients (1). Due to the rarity of these cases, the natural history is not well understood. Steroid use for the treatment of optic neuritis has been implicated in the development of subsequent retinitis [8].

Based on the timeline of development of symptoms in our patient, we suspect that she had viral meningitis and subsequently went on to develop a right optic neuritis (optic nerve), left CRAO (retinal artery), and bilateral PORN (retina) in a contiguous manner. Another plausible explanation is that of the virus causing vasculitis of the posterior ciliary artery and retinal vasculature. CRAO developing approximately a month later after the initiation of HAART (highly active antiretroviral therapy) could also be a manifestation of immune reconstitution syndrome. We believe that prompt administration of HAART, aggressive management of CRAO, and administration of both systemic and intraocular ganciclovir and systemic cidofovir were factors responsible for the good visual recovery in our patients. Therefore, we recommend considering therapeutic interventions even in patients with almost complete visual loss.

## 4. Conclusion

- VZV infection can be seen in every stage of HIV. Reactivation of VZV in the trigeminal ganglion can lead to the various ophthalmological manifestations.
- Sudden onset visual loss in HIV patients could be secondary to optic neuritis, retinitis, or retinal artery occlusion.
- Optic neuritis is typically seen after herpes zoster skin infection. Clinically, it manifests as loss of visual acuity and afferent pupillary defect. It has been known to precede PORN.
- PORN occurs in severely immunocompromised patients and is rapidly progressive. It is characterized by rapidly progressive lesions and has an extremely poor prognosis.
- Retinal artery occlusion can be seen in association with VZV and could be due to either direct invasion or granulomatous angiitis of the arterial wall.
- Prompt administration of HAART, aggressive management of retinal artery occlusion, and administration of both systemic and intraocular ganciclovir and systemic cidofovir were factors responsible for the good visual recovery in our patient. Therefore, we suggest that even patients with almost complete visual loss should get therapeutic interventions.

## Figures and Tables

**Table 1:** Laboratory assessment.

CSF WBC (0–5 cells/microlt)	135
CSF lymphocytes	129
CSF glucose (40–80 mg/dl)	97
CSF protein (15–45 mg/dl)	<2
CD4 count (500–1500)	10
HIV viral load (<50 copies/ml)	184,530
Cryptococcal antigen	Negative
Herpes simplex virus PCR	Negative
Cytomegalovirus PCR	Negative

^*∗*^Note: microlt: microliter; mg: milligram; dl: deciliter; ml: milliliter.

**Table 2:** Ophthalmic manifestations of varicella zoster.

Eyelids	Entropion, trichiasis, epicanthoid lesions, paralytic ptosis
Conjunctiva and sclera	Follicular conjunctivitis, scleritis
Cornea	Punctate epithelial keratitis, stromal keratitis, neurotrophic keratitis
Uveal system	Iritis, Argyll Robertson pupil, phthisis bulbi, glaucoma
Retina	Acute retinal necrosis, posterior outer retinal necrosis, central retinal artery occlusion, central retinal vein occlusion
Extraocular muscles	Extraocular muscle paralysis
Optic nerve	Optic neuritis

Source: Ref. [7].
